# Adolescent girls, a forgotten population in resource-limited settings in the COVID-19 pandemic: implications for sexual and reproductive health outcomes

**DOI:** 10.11604/pamj.supp.2020.37.1.26970

**Published:** 2020-11-25

**Authors:** Grant Murewanhema

**Affiliations:** 1 Department of Obstetrics and Gynaecology, University of Zimbabwe College of Health Sciences, PO Box A178, Avondale, Harare, Zimbabwe

**Keywords:** COVID-19, adolescents, sexual, reproductive, health, gender-based, violence, genital, mutilation

## Abstract

Adolescent sexual and reproductive health is an essential aspect that may be forgotten in the COVID-19 pandemic. Valuable insights gained from previous humanitarian crises indicate undesirable short and long-term adolescent maternal consequences in low resource settings. Young girls are at a higher risk of dropping out of school and being forced into early child marriages and high-risk jobs that predispose them to sexual exploitation and sexual and gender-based violence. Economic recessions, supply chain disruptions and reallocation of resources may limit access and utilisation of services and commodities. The COVID-19 pandemic thus indirectly exposes adolescent girls to multiplied risks of unintended pregnancies, sexually transmitted infections including HIV and Human Papilloma Virus. Sexual and gender-based violence, including female genital mutilation cases may increase as intervention programmes to avert these are disrupted, and the resultant psychosocial and socioeconomic consequences may be devastating. Thus, a pro-active approach is required to come up with frameworks to ensure the minimum initial service package for reproductive health. A multi-sectoral collaborative intersection of relevant stakeholders in adolescent sexual and reproductive health is therefore urgently desired.

## Perspectives

The burden of the global COVID-19 pandemic continues to grow and now exceeds 21 million confirmed cases [1]. The World Health Organisation (WHO) situation reports indicate that 1.7 million new infections were reported in the week ending 24 August 2020 [1]. As fatigue and complacency sets in, the battle with the pandemic is likely to be protracted. Previous disasters and experiences indicate key populations that may be neglected and suffer disproportionately from the indirect effects of adversity times [2]. Estimating the indirect impacts of the pandemic may be difficult, and confounded by poor surveillance systems, especially in low-resource settings. However, a pro-active approach to mitigate against undesirable consequences is desirable, necessitating the need for drawing frameworks that identify and prioritise vulnerable groups. Tang *et al*. note that sexual and reproductive health (SRH) issues are key in the COVID-19 response [3]. SRH matters for adolescent girls are forgotten in humanitarian crisis times [4], with undesirable short and long-term implications, particularly in the low-resource settings of sub-Saharan Africa (SSA). The WHO defines adolescents as persons between 10-19 years. However, research often extends the definition to include persons up to 24 years of age and lumps them together as young people. In a systematic review by Jennings *et al*. it was noted that adolescent health, including SRH, is often neglected in health emergency settings [5]. Previous observations estimated 32 million young women and girls to be living in humanitarian situations, with an increased unmet need for SRH services [6]. This number could be much higher in the global COVID-19 pandemic.

In SSA and other resource-limited settings, SRH programmes are usually development partner supported. Reprioritisation of COVID-19 response activities may result in resource re-allocation, including funds and personnel. COVID-19 preventive interventions including lockdowns and travel restrictions potentially can result in reduced access and utilisation of SRH services by adolescent girls. On the other hand, school closures mean increased time spent in the community, exposing young girls to sexual activities, sexual exploitation and sexual and gender-based violence (SGBV). I review the implications of the COVID-19 pandemic on SRH for adolescent girls in low-resource.

### Sexual and reproductive health for adolescent girls in emergency settings

**Early child marriages:** in a scoping review of SRH for Syrian refugees in Turkey, early marriage, low modern contraceptive use, unmet need for contraception and SGBV were frequently reported, with a mean age of marriage of 18-20 years across several studies [7]. Due to differential prioritisation still rife in some parts of SSA, girls are 2.5 times more likely to drop out of school than their male counterparts do [8]. The COVID-19 pandemic may result in an additional 13 million child marriages between 2020 and 2030 owing to socioeconomic degeneration and disruption of plans to end child marriages [9]. As marginalised communities struggle for upkeep, adolescent girls are forced into early marriage [9]. Religious beliefs and bride price issues complicate the situation. Being married to older men and polygamous marriages increases the risk of HIV and Human Papilloma Virus (HPV) acquisition [10]. Evidence from SSA settings shows patriarchy and bridal price as important drivers of the HIV pandemic, and early acquisition of HPV is a risk factor for the development of cervical cancer [11]. This comes at a time when HPV vaccination programmes may be disrupted due to stoppage of essential health services and global disruptions of supply chains. HPV vaccination is efficacious for primary prevention of cervical cancer, more so in low-resource settings where screening programmes may be limited and the risk of cervical cancer is considerable. Dropping out of school may mean seeking of high-risk jobs including vending and cross-border trading which expose adolescent girls to sexual exploitation, SGBV and multiple concurrent partnerships, all risk factors for HIV, HPV and other sexually transmitted infections (STIs) and unintended pregnancies [12].

**HIV prevention:** access to HIV prevention services may be reduced. Access to condoms and lubricants may decrease due to supply chain disruptions and development partners' changed focus and resource allocations towards containing the COVID-19 pandemic [13]. Owing to patriarchy, the adolescent girls and women may not have the power to negotiate the use of condoms [14], and the frequency of unprotected coitus is higher in this age group. In an ethnographic study in rural Zimbabwe, Duffy L noted women's oppression through gender inequality as a recurring theme and contributory factor towards HIV acquisition [15]. School closure and movement restrictions are potential barriers of access to critical health promotion messages that reinforce the need to stay away from early sexual activities. UNAIDS statistics estimate 5500 women aged 15-24 years to contract HIV weekly [16]. In SSA, five out of every six new infections among adolescents aged 15-19 years occur in girls [16]. The COVID-19 pandemic may aggravate the situation, and this population remains key for control of the HIV epidemic in Africa.

**Sexual and gender-based violence:** reports of increased SGBV emerged in the Ebola Viral Disease (EVD) outbreaks in West Africa, and have started to emerge in the COVID-19 pandemic [17]. Disaster times exaggerate pre-existing societal power structures and gender inequities [17]. SGBV undermines the health, dignity, security and autonomy of its victims. Projections estimate that a 20% increase in violence during lockdown periods could result in an additional 15 million cases of SGBV for every three additional months of lockdown, globally [9]. A considerable amount of SGBV cases will occur among adolescents. Amnesty International reports indicate that even security forces abused girls and young women in quarantine centres [17]. Quarantine centres have been set up to house returning residents from COVID-19 transmitting regions in a number of SSA countries. With reduced access to services for rape survivors, which include HIV and STI Post-exposure prophylaxis and emergency contraception, there may be increased incident STIs and HIV infections, and unintended pregnancies. Unintended pregnancies tend to increase the risk of unsafe abortions, with consequent rises in septic miscarriages, haemorrhage and maternal morbidity and mortality. Scholarly mathematical models show that reduced access to regular and emergency contraception may considerably increase adverse maternal and neonatal outcomes.

**Contraceptive access:** a huge unmet need for contraception existed before the COVID-19 pandemic in some low-resource countries, and may worsen during and beyond the pandemic. The United Nations Population Fund (UNFPA) estimated the unmet need in Zimbabwe to be 12.6% in the 15-19 year old group before the onset [18]. This is higher than the unmet need in the general population, which stood at 10.4% [18]. Closure of family planning clinics, increased costs and reduced availability stemming from supply chain disruptions owing to reduced production and delayed movements may limit contraceptive availability, especially for marginalised communities [9]. Reduced contraceptive coverage of 10% over a year could add 15 million unintended pregnancies, with an additional 28 000 maternal and 168 000 neonatal deaths. In West Africa, maternal mortality rose by 70% during an EBV outbreak [19]. Similarly, a 10% reduction in access to safe abortions could result in 3.3 million unsafe terminations and one thousand maternal deaths [4].

**Teenage pregnancies:** higher incidence of teenage pregnancies was reported in the EVD outbreak in Sierra Leone [20]. Teenage pregnancies are high-risk pregnancies. According to the WHO, complications during pregnancy and delivery are the leading cause of death for the 15-19-year-old girls globally. Adolescent mothers aged 10-19 years have higher risks of pre-eclampsia, puerperal sepsis and post-partum endometritis, and their babies are at a higher risk of preterm delivery, low birth weight and severe neonatal complications, compared to women aged 20-24 years. Adolescents conceiving in the COVID-19 pandemic may be at increased risk of adverse maternal and neonatal outcomes due to disruption of maternity care [21]. Antenatal surveillance is reduced or absent, and maternity units for labour and delivery are operating sub-optimally. Long-lasting consequences of obstructed labour and delayed intervention include urogenital fistulae, which dramatically alter the course of life for many women in poor communities. Socioeconomic consequences of teenage pregnancies include rejection by families, seeking high-risk jobs for survival, including turning to commercial sex work for survival, and unstable families headed by children. Return to education after teenage pregnancies is uncommon in the developing world, and is complicated by the prevailing harsh economic conditions in most of SSA [18]. The young mothers, who may be rejected by the fathers of their children, often have to seek child employment, and in countries with high unemployment rates, this is usually high-risk informal employment, which exposes to further sexual exploitation. Without adequate social insurance and support, a vicious cycle may erupt with repeated unintended conceptions, unsafe terminations, HIV, STIs and reduced life expectancy.

**Female genital mutilation:** an estimated 200 million young women and girls have been forced to undergo female genital mutilation (FGM) [9]. FGM is often a precursor to early forced marriages, which ends a girl's education and dims her prospects of a brighter future [9]. The COVID-19 pandemic could introduce delays in programmes for averting FGM. UNICEF and the UNFPA note that an estimated two million cases of FGM will need to be averted during the COVID-19 pandemic [22]. As social and security services are disrupted and not prioritised in emergency settings, adolescents fail to find appropriate interventions timeously and may have nowhere to report these abusive societal tendencies.

**Long-term consequences:** the psychosocial effects of SGBV, FGM and rape can be long standing and include failing to cope with pressures of life, post-traumatic stress disorder, depression, suicide and failed relationships [23]. Feelings of inferiority, mistrust, fear, panic attacks, substance misuse, sleep disturbances, eating disorders and psychosexual dysfunction are recognised complications [23]. Lack of immediate psychosocial counselling post potentially traumatic SRH events has longstanding repercussions for the adolescent girls. Stigma, isolation and rejection, loss of potential income and homicide tendencies such as female infanticide can be resultant social ills [23]. Long-term physical health outcomes include subfertility, cervical cancer and HIV/AIDS among others [23]. Thus, proactively setting up frameworks to protect adolescent girls as the COVID-19 pandemic persists, based on experiences from the past, and must be urgently prioritised.

**Recommendations:** the United Nations High Commission for Refugees (UNHCR) recognised the need to develop a coordinated set of priority activities to prevent and manage the consequences of SGBV, reduce HIV transmission, prevent excess maternal (and newborn) morbidity and mortality, and plan for comprehensive reproductive health (RH) services [24]. These are termed the Minimum Initial Service Package (MISP) for RH. Additional priority activities of the MISP encompass increasing availability of contraceptives to meet demand, syndromic management of STIs, and ensuring antiretroviral (ARV) availability for continuing users [24]. Based on the MISP, frameworks for restoring and maintaining SRH for adolescent girls, to protect them from unfavourable short and long-term sequelae must be drawn. As more information on COVID-19 emerges, governments must start redirecting efforts towards restoration of essential health and social services at facility and community level, in environments that protect healthcare practitioners and clients from contracting COVID-19. Contextualised guidance on maintaining essential health services, HIV prevention services, family planning and other components of SRH for adolescent girls can be developed from guidance from WHO, UNFPA, UNICEF and other development agencies. Understanding local COVID-19 transmission dynamics is critical for developing suitable guidelines for settings, thus governments must strengthen surveillance for COVID-19, and integrate surveillance for indirect impacts of the COVID-19 pandemic to rationalise the utilisation of scarce commodities, financial and human resources. A multi-disciplinary approach involving health practitioners, educationists, social and security services, women empowerment groups and key community stakeholders may yield the best results. To this end, wider consultative forums must be prioritised to define the most effective ways of restoring essential SRH services for adolescent girls drawing on the MISP.

## Conclusion

Lessons from prior humanitarian crises must remind us not to neglect adolescent girls' health, a population at risk of adverse SRH outcomes, with short-term and long-term undesirable consequences. A reflection of the experiences in the COVID-19 pandemic so far is critical as an effective way forward is mapped to mitigate adverse adolescent SRH sequelae. A multi-sectoral collaborative approach is critical to optimising restoration, continuity and outcomes of essential adolescent SRH during and beyond the COVID-19 pandemic.
